# An integrative morpho-molecular approach in malignant ectomesenchymoma diagnosis: report of a new paediatric case and a review of the literature

**DOI:** 10.3389/fonc.2024.1320541

**Published:** 2024-03-01

**Authors:** Francesco Pellegrino, Elisa Tirtei, Federico Divincenzo, Anna Campello, Carlotta Rubino, Elisabetta Augustoni, Alessandra Linari, Sebastian Dorin Asaftei, Franca Fagioli

**Affiliations:** ^1^ Department of Pediatric and Public Health Sciences, Postgraduate School of Pediatrics, Regina Margherita Children Hospital, University of Turin, Turin, Italy; ^2^ Department of Public Health and Paediatrics, University of Turin, Turin, Italy; ^3^ Paediatric Onco-Haematology, Stem Cell Transplantation and Cellular Therapy Division, Regina Margherita Children’s Hospital, City of Health and Science of Turin, Torino, Italy; ^4^ Department of Pathology, Azienda Ospedaliera Città della Salute e della Scienza, Turin, Italy

**Keywords:** malignant ectomesenchymoma, soft tissue tumor, morpho-molecular analysis, pediatric oncology, next generation sequencing

## Abstract

**Introduction:**

Malignant ectomesenchymoma (MEM) is a soft tissue tumour, consisting of both malignant neuroectodermal elements and one or more mesenchymal elements.

**Case presentation and review of the literature:**

Here we describe the case of a 6-months-old male, previously treated in another hospital for abdominal rhabdomyosarcoma (RMS). Histological re-examination demonstrated that the tumour had mesenchymal and neuroectodermal elements components, with a new diagnosis of abdominal-pelvic MEM. A Next-Generation Sequencing (NGS) analysis was performed on a surgical tumour specimen and revealed the presence of a somatic mutation, already reported in MEM cases. We carried out a review of the literature and we found 33 new cases of MEM since the last review. We reported the clinic-pathologic features of new cases of MEM, highlighting the role of molecular studies in supporting the diagnosis of this ambiguous tumours.

**Conclusion:**

We promote the importance of a diagnosis based on an integrative morpho-molecular approach, that routinely include molecular analysis and the use of bioinformatic mutation detection tools, to support diagnostic and therapeutical queries and to highlight tumour biology and behaviour.

## 1 Background

Malignant ectomesenchymoma (MEM) is an uncommon and rapidly progressing soft tissue tumour, composed of both malignant mesenchymal and neuroectodermal elements (1–3). The predominant mesenchymal elements are usually embryonal rhabdomyosarcoma (ERMS), while the neuroectodermal component may present with multiple degrees of differentiation, emerging as neuroblastoma (NB), ganglioneuroma (GN), ganglioneuroblastoma (GNB) or ganglion cells (GC). Most commonly the neural component occurs as clustered GC and, rarely, as primitive neuroblastic elements. Sporadic cases of alveolar rhabdomyosarcoma (ARMS), malignant peripheral nerve sheath tumour, and primary peripheral neuroectodermal tumour have been reported (4).

MEMs are presumed to originate from the remnants of migratory cells of the neuronal crest, which constitute the ectomesenchyme (5). These pluripotent cells are widespread throughout the body; thus MEMs may potentially develop at any site within the soft tissue or in the central nervous system. The most common primary sites include pelvic and retroperitoneal region and urogenital sites, whereas a less often they arise in the head and neck region or mediastinum (3). The exact aetiology is unknown.

It is commonly assumed that MEM patients should be treated according to RMS protocols, as the risk factors, treatment and outcomes of these tumours are comparable to other highly malignant paediatric soft tissue tumours, such as ERMS (6, 7).

More than 60 cases of MEM have been reported in the literature, predominantly concerning young children and adolescents. Here we expand the knowledge of this rare tumour, by describing the clinicopathologic spectrum of the 22 cases that have not been previously reported as well as reviewing the previously reported cases, highlighting the supportive role of immunohistochemistry and molecular analysis for diagnosis.

## 2 Case report

The patient was a 6-month-old male infant, treated in another hospital for abdominal rhabdomyosarcoma (RMS). The patient was treated according to EpSSG (European paediatric Soft tissue sarcoma Study Group) protocol, with 4 IVADo (Ifosfamide, Vincristine, Actinomycin, Doxorubicin) cycles (8). In the post-chemotherapy Magnetic Resonance Imaging (MRI), the patient showed a stable disease according to Response Evaluation Criteria in Solid Tumours (RECIST v1.1) (9).

**Figure 1:**
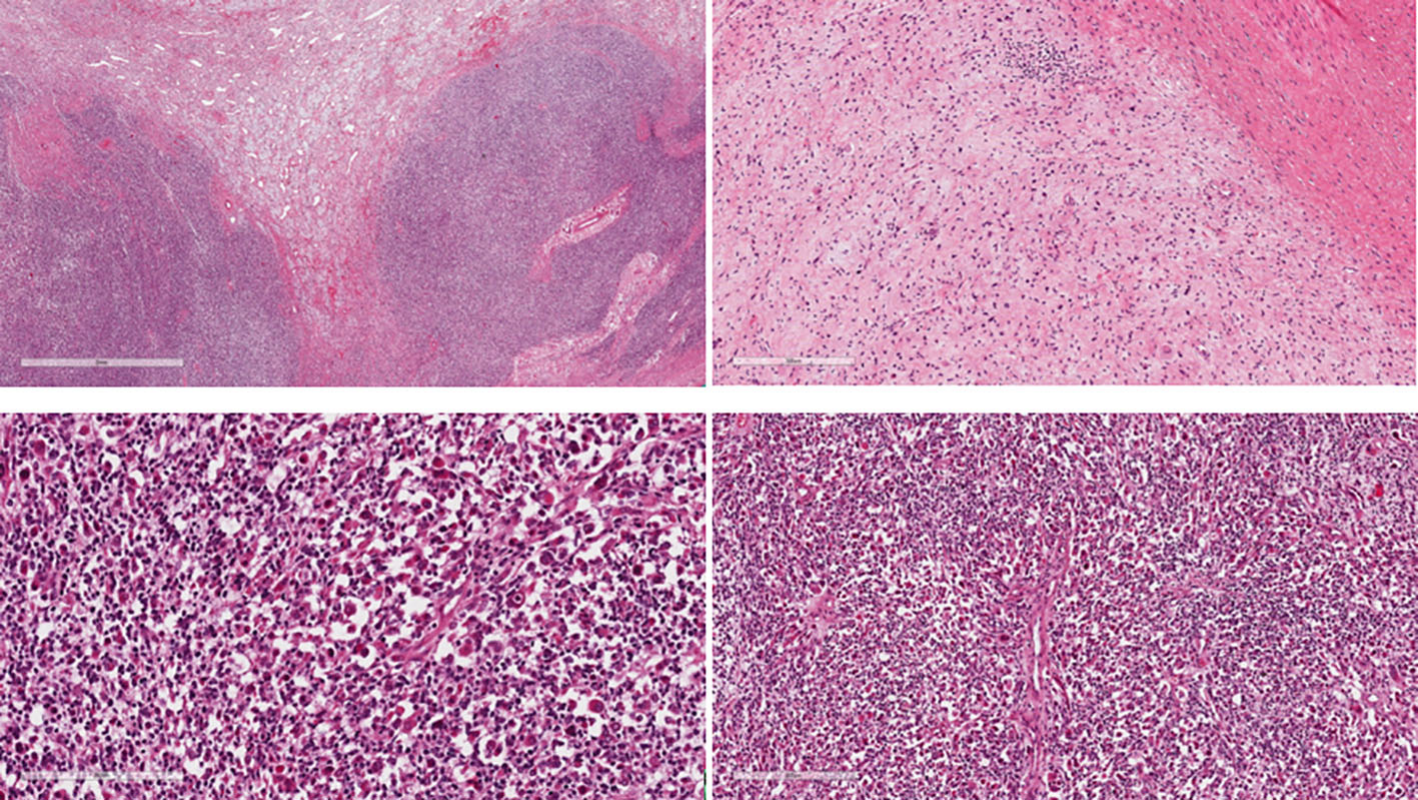
The tumour shows biphasic atypical cells composed of mesenchymal and neuroectodermal elements predominantly intermingled but also with distinct borders at places.

Two more chemotherapy cycles were performed according to the VIT (Vincristine, Irinotecan and Temozolomide) regimen (10), and the subsequent radiological evaluation showed a stable disease (SD) according to RECIST 1.1 with a mild tumour volume reduction, inferior to 20%. Then he was referred to our hospital, where radical excision of the tumour was performed without surgical complication. Histological examination demonstrated that the tumour presented two different elements. The main component was composed by mesenchymal and neuroectodermal elements. The first element consisted of spindle cells, similar to rhabdomyosarcomatous elements. Immunoistochemical staining showed positivity for desmin and myogenin. Thus, ERMS was considered. The tumour’s neuroectodermal element showed ganglion-like cells, positive for synaptophysin, so ganglioneuroma was considered. Based on the pathological findings, a malignant ectomesenchymoma suspected (**Figure 1**).

A Whole Exome Sequencing (WES) analysis was performed on a surgical tumour specimen with Illumina® DNA platform to detect DNA tumour mutations. The analysis revealed the presence of somatic mutation *HRAS : NM_176795:exon2:c.G37C:p.G13R* (Variant Allele Frequency (VAF):72%), already reported in MEM cases (3). No mutations of FOX1 were detected.

*HRAS G13R* is hotspot mutation that lies within the Guanosine-5’-triphosphate (GTP) binding domain of Harvey Rat Sarcoma Virus Oncogene (*HRAS*) protein, and results in activation of Mapk and Pi3k signalling (11). G13R mutation predicts a loss of *HRAS* protein function (11).

The patient received two more VIT regimen cycles as postoperative chemotherapy. Then, a computer-tomography (CT) was performed, which showed a stable complete remission. Therefore, the patient was discharged and prescribed maintenance chemotherapy with Cyclophosphamide and Vinorelbine for 12 months (12).

A first radiological evaluation with CT was performed 3 months after starting maintenance, and a complete remission was confirmed. However, the radiological evaluation performed 6 months after the beginning of maintenance treatment, showed a rectal tumour mass at the scar site of the previous surgery. A complete staging was performed with MRI and a whole-body Positron Emission Tomography (PET)-CT scan to exclude any distant metastasis. Due to the location of the tumour, which infiltrated nearby organs (rectum, bladder, and prostate), surgeons performed a mass debulking because a complete resection was not feasible. Then, two chemotherapy courses following a Topotecan and Cyclophosphamide regimen were administered (13). Approximately 2 months after tumour recurrence, the patient’s clinical condition worsened, and he passed away.

The mesenchymal component shows intersecting fascicles of pleomorphic spindle cells similar to rhabdomyosarcomatous elements. The cells present vesicular nuclei and a scant amount of eosinophilic cytoplasm.

The neuroectodermal component is arranged in loose irregularly oriented bundles with isolated ganglion-like cells with copious eosinophilic cytoplasm.

Iimmunohistochemistry: RMS cell: anti-desmin and anti-myogenin positive. GN cells: anti- Synaptophysin and S-100 positive (images not available).

## 3 Materials and methods

In this retrospective study, we conducted a systematic literature search of MEDLINE, EMBASE and Pubmed databases to identify studies describing cases of MEMs. The search and selection of articles was carried out in accordance with PRISMA guidelines.

No restrictions were imposed on the language and type of studies, including case reports that described patients with MEMs. All human patients with a confirmed histological diagnosis of MEM, with no restrictions on age or other demographics, were included.

**Figure 2:**
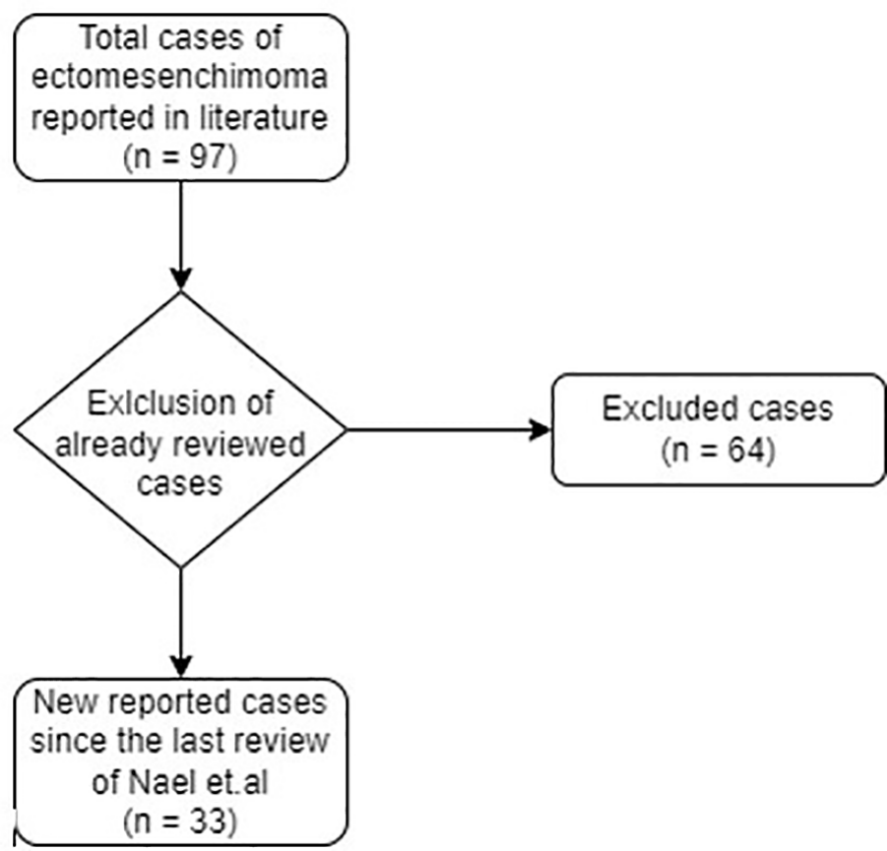
Selection of new MEMs cases, reported in literature since the last review of 2014.

The following data were collected from the studies retrieved: first author, year of publication, type of article, number of cases described, sex and age of the patients, past medical history, clinical manifestation at onset, disease site radiographic imaging, histology and immunohistochemistry, type of treatment (surgery, medical therapy, clinical and/or imaging surveillance), follow-up, outcome. The databases research produced a total of around 90 literature articles on MEMs. Applying the filters to select articles subsequent to the last review of Nael et al., and after application of PRISMA guidelines, 12 article were retrieved, for a total of 33 new cases (**Figure 2**).

## 4 Results

Including our case, 98 MEM cases were described in literature. Freitas et al. initially reported 40 MEM cases from 1946 to 1998 (14), while Nael et al. described a further 24 MEM cases, from 1998 to 2014, with related data regarding gender, age, primary site, histology pattern, treatment, and survival of patients since the time of presentation (15). After reviewing the literature from 2014 to present, we found another 33 MEM cases. The clinic-pathologic features of new reported cases of MEM and our new case are summarised in **Table 1**.

**Table 1:** Cases of malignant ectomesenchymoma reported in literature after the last Review of Nael et al. in 2014.

Cases Reference	Age	Sex	Primary site	Recurrence of metastasis	Histology	Immuno-histochemistry	Treatment	Molecular alterations	Follow-up
VandenHeuvel A. (2014) (16)	22 mo	F	Tongue	No	RMS + GN	RMS: myogenin positive in 50% and desmin positive in 30% of cells; GN: S-100 pos.	TSR + CT	*PAX3-FOXO1* and *PAX7-FOXO1* gene fusion negative	NED after 7 months
Kao W. (2015) (17)	34 years	M	Paratesticular (left)	Lung metastasis after 2 weeks from operation	Anaplastic embryonal sarcoma + GN	AES: desmin, myogenin, myoglobin pos; GN: synaptophysin pos. SC: S-100 pos.	TSR + CT	NA	NA
Kun Y. (2015) (18)	4 years	F	Left fronto-parietal lobe	No	Mesenchymal region composed of spindle-cells + Anaplastic ependymoma and astrocytoma	Mesenchymal component: reticulin and INI-1 pos. Desmin and SMA neg. Anaplastic ependymoma: GFAP, nestin, MAP-2, vimentin and EMA pos. Olig-2 and S-100 partially pos. Astrocytoma: GFAP, S-100 and vimentin pos.	TSR + CT	10 q deletion and EGFR gene amplification negative	NED after 5 months
Huang S. (2018) (3)	0.6 mo	M	Paratesticular	NA	ERMS + GC	ERMS cell: anti-desmin and anti-myogenin pos. GG cells: anti- Synaptophysin and S-100 pos	TSR + CT	HRAS mutation positive PTPRD or FBXW7 negative	NED at 8 years
	12 mo	M	Paratesticular	NA	ERMS + GN	ERMS cell: anti-desmin and anti-myogenin pos. GN cells: anti- Synaptophysin and S-100 pos	NA	HRAS mutation positive PTPRD or FBXW7 negative	NED at 2 years
	8 mo	M	Urinary bladder	NA	ERMS + GN	ERMS cell: anti-desmin and anti-myogenin pos. GN cells: anti- Synaptophysin and S-100 pos	TSR + CT	HRAS mutation positive PTPRD or FBXW7 negative	NA
	3 mo	M	Pelvis	NA	ERMS + GN	ERMS cell: anti-desmin and anti-myogenin pos. GN cells: anti- Synaptophysin and S-100 pos	TSR + CT	HRAS mutation positive PTPRD or FBXW7 negative	DOD at 0.9 years
	11 mo	M	Pelvis	NA	ERMS + NB	ERMS cell: anti-desmin and anti-myogenin pos. NB cells: anti- Synaptophysin and S-100 pos	TSR + CT	HRAS negative PTPRD or FBXW7 negative	NED at 2.9 years
	1.2 mo	M	Prostate	NA	ERMS + GN	ERMS cell: anti-desmin and anti-myogenin pos. GN cells: anti- Synaptophysin and S-100 pos	TSR + CT	HRAS mutation positive PTPRD or FBXW7 negative	NED at 8.1 years
Glaser A.P. (2017) (19)	15 years	M	Paratesticular	NA	RMS + Unknown neuroectodermal component	NA	TSR + CT	NA	NED at 15 months
Griffin BB. (2018) (4)	4 years	F	Right axilla	NA	ARMS + NB	ARMS and NB: desmin, myogenin, synaptophysin positive.	NA	PAX3-FOXO1 fusion product detected by RT-PCR	NA
	11.5 mo	M	Perineum	Locoregional lymph node metastasis	ARMS + NB	ARMS and NB: desmin, myogenin, synaptophysin positive.	CT	PAX3-FOXO1 fusion product detected by RT-PCR FISH studies negative for FOXO1 rearrangement	NA
	6 mo	F	Right posterior neck	NA	ARMS + pPNET	ARMS: desmin, myogenin pos. pPNET: desmin, myogenin and synaptophysin pos.	PRS + CT	PAX7-FOXO1 fusion product detected by RT-PCR	Alive with met.
	15 years	M	Right para-testicular area	NA	ERMS + GN	ERMS: desmin, myogenin pos. GN: S-100 and synaptophysin pos.	PRS + CT	No fusion/alteration by Archer FusionPlex Sarcoma gene panel	NED
	1 mo	F	Perineum	NA	ERMS + GN	ERMS: desmin, myogenin pos. GN: S-100 and synaptophysin pos.	PRS + CT	NA	NED
	4 mo	F	Left posterior neck	Brain metastasis	ERMS + pPNET	ERMS: desmin, pos. pPNET: rare synaptophysin pos.	PRS + CT	No fusion/alteration by Archer FusionPlex Sarcoma gene panel	DOD
Boudjemaa S. (2019) (20)	17 mo	M	Tongue	No	ERMS + GC	ERMS: Desmin and myogenin pos. GC: Synaptophysin, chromogranin and S100 pos	PRS + CT	NA	NED at 6 years
Kung H. (2021) (21)	4 mo	M	Inguinal	No	ERMS + GC	ERMS: myogenin, desmin, myo-D1 pos GC: S100, Synaptophysin and SOX10 pos	TSR + CT	NA	NED at 2 years
	6 mo	F	Inguinal	No	RMS + GC	RMS: myogenin, desmin, myo-D1 pos GC: S100, Synaptophysin and SOX10 pos	TSR + CT	NA	NED at 6 months
Rashid T. (2021) (22)	7 mo	M	Eye orbit	No	RMS + GN	RMS cell: anti-myogenin pos. GN cells: anti- Synaptophysin POS	CT + local RT during CT	TP53 negative	NED at 6 years
Davidson B (2021) (23)	72 years	F	Pelvic	Locoregional lymph node and small bowel, pulmonary nodules	Spindle cell + GC	Spindle cell: Vimentin and CD10 pos GC: Synaptophysin SALL4 pos	CT	c.5428G>T; p.Asp1810Tyr DICER1 missense mutation missense mutation in TP53 (c.730G>A; p.Gly244Ser) missense mutations in PTEN (c.376G>A; p.Ala126Thr and c.406T>C; p.Cys136Arg). No fusion genes were detected	Alive with met.
Hernández-Reséndiz (2022) (24)	23 years	M	Pineal region	No	Spindle cells + Rhabdomyoblasts + GC	Rhabdomyoblasts: calcium-binding, protein B S-100B, myoglobin, myogenin, and actin pos GC: eu-N, synaptophysin, neuron-specific enolase (NSE), and S-100 pos	TSR	NA	NA
Pena-Burgos (2023) (25)	15 years	F	Right parapharyngeal - Neck region	No	ERMS + GC	ERMS: MyoD1 pos, focally cytoplasmic positive for desmin and nuclear positive for myogenin GC: neuronal nuclear antigen (NeuN), neurofilaments and synaptophysin pos	CT	p.Leu122Arg (c.365 T > G) mutation in *MYOD1* gene p.Ala34Gly mutation in *CDKN2A* gene *CDK4* gene amplification Translocation of the *FOXO1* and *ETV6* gene negative	DOD after 17 months
Milano (2023) (26)	4 mo	F	Pelvis	No	ERMS + GC	GC S100 pos	CT + SR	FOXO1 negative	Alive in first remission
	5 mo	M	Paratesticular	No	ERMS + GC	GC S100 pos	CT + SR	FOXO1 negative	Alive in first remission
	7 mo	M	Bladder - Prostate	No	GC	NA	CT + SR + RT	FOXO1 negative	Alive in first remission
	17 mo	M	Paratesticular	No	ERMS + GC	NA	CT + SR	FOXO1 negative	Alive in first remission
	13 mo	F	Vagina	No	Small round cell component co-expressing	S100 and myogenin pos	CT + SR	FOXO1 negative	Alive in first remission
	2 years	M	Paratesticular	No	ERMS + GC with schwannian component	NA	CT + SR	FOXO1 negative	Alive in first remission
	3 years	M	Abdominal wall	Locoregional lymph nodes	ERMS + GC with schwannian component ERMS + GC with schwannian component	NA	CT + SR + RT on primary tumour and regional lymph nodes after progression	FOXO1 negative	Alive in second remission, 3 years from relapse
	6 mo	M	Bladder-Prostate	Locoregional lymph nodes and lungs	ERMS + GC with schwannian component	NA	CT + SR	FOXO1 negative	DOD 16 months after first diagnosis
	4 years	F	Orbit	No	ERMS + GC with schwannian component	NA	CT + SR + RT	FOXO1 negative	Local relapse 8 months from diagnosis, alive in second remission 3 years from relapse
	6 years	F	Parotid gland	No	ERMS + GC	GC S100 pos	CT + SR + TR	FOXO1 and TP53 negative	Secondary acute myeloid leukaemia after 15 months off therapy, alive in remission from both tumours more than 10 years after MEM
Pellegrino (2023)	6 mo	M	Abdomen - Pelvic	Sacral lymph node	ERMS + GN	RMS cell: anti-desmin and anti-myogenin pos. GN cells: anti- Synaptophysin and S-100 pos	SR + CT	HRAS G13R mutation positive	DOD at

ARMS, alveolar rhabdomyosarcoma; CT, chemotherapy; DOD, dead due to disease; EMA (epithelial membrane antigen); ERMS, embryonal rhabdomyosarcoma; F, female; GFAP glial fibrillary acidic protein; GC, ganglion cell; GN, ganglioneuroma; GNB, ganglioneuroblastoma; M, male; Met., metastasis; mo., month(s); NA, no data available; NB, neuroblastoma; NED, no evidence of disease; pPNET, peripheral primitive neuroectodermal tumour; RMS, rhabdomyosarcoma; RT, radiation therapy; TSR, total surgical resection; SR, surgical resection.

Thirteen (38%) of these are females and 21 (62%) are males. The age at diagnosis ranged from 0.6 months to 72 years, with a median age of 12 months. Eighteen patients (53%) developed the tumour in the first year of life (3, 4, 21, 22, 26).

The most common tumour localisation was the genitourinary/pelvic region (23 cases, 67%) (3, 4, 17, 19, 21, 23, 26). ERMS was the prevalent mesenchymal component in the majority (21/34) of tumours (3, 4, 20, 21, 25, 26).

Immunohistochemical analysis revealed diffuse positivity in ERMS-like mesenchymal elements for desmin and myogenin, while neuroectodermal elements (GNB, GN e GC) presented a diffuse positivity for synaptophysin and S-100. Interestingly, in cases of MEMs with ARMS-like and NB elements, both type of cells presented diffuse positivity for desmin, myogenin and synaptophysin.

Seventeen patients received primary total surgical excision of the tumour (3, 16–19, 21, 24), while 11 underwent partial surgical excision (4, 20, 26), in both cases followed and/or preceded by chemotherapy. The chemotherapy regimen was the same used for RMS which included ifosfamide, vincristine, actinomycin-D (IVA) or ifosfamide, vincristine, actinomycin-D, doxorubicin (IVADo) for nine courses, plus surgery and/or radiotherapy according to the risk of local failure (27).

Radiotherapy (RT) was performed in five patients (22, 26).

Locoregional lymph-nodes metastases were reported in five patients (4, 23, 26) and four patients also had distant metastatic disease. The most common site of distant metastasis were lungs (75%). Distant metastasis was always detected at first diagnosis, except for one patient with lung metastasis, in whom they were detected after 2 weeks from surgery. The presence of distant metastasis is more common in patients with abdominal/pelvis MEM and is usually associated with a worst prognosis.

Griffin et al. detected rearrangements of the *FOXO1* gene in three cases with alveolar RMS morphology, including two with *PAX2-FOXO1* fusion, and one *PAX7-FOXO1* translocation (4).

Moreover, Huang et al. studied seven MEMs by RNA sequencing and found *HRAS*, *PTPRD* and *FBXW7* mutations respectively in six, two and one cases. No fusion genes were reported. They observed oncogenic mutations in *RAS* signalling pathway also in the control paediatric ERMS. The *HRAS* mutations detected in the MEMs case described by Huang, were identical to 3 ERMS cases reported in literature (3). Furthermore, Davidson and co-workers sequenced the paraffin-embedded tissue (FFPE) material from the patient’s lymph node metastasis, containing both neoplastic cellular elements, in which they reported the presence of missense mutations in *DICER1, TP53* and *PTEN* (23). However, molecular studies of MEMs are so far restricted to these few reports (3, 4, 23).

Follow-up data are available for 28 patients (82%), of whom 23 were surviving with no evidence of disease (NED) following multimodality treatment approach, while 4 died due to disease (DOD) and 1 was alive with metastasic disease. Follow-up period ranged from 5 to 72 months, with an average of 38.5 months. Five patients were lost to follow-up.

## 5 Discussion

Malignant ectomesenchymoma (MEM) is an extremely rare tumour, with only about 75 cases having been reported to date (14). The last review was conducted by Nael et al. in 2014 (15). Therefore, the aim of this review is to update the epidemiologic and clinical data about this rare disease, to improve our still limited knowledge of biological behaviour, histological characteristics, treatment and prognosis.

MEMs present most commonly in children, primarily involving infants during the first year of life (14). The most common anatomical site is pelvic and abdominal region, followed head and neck, and mediastinum (3). Our review shows a male-to-female ratio of 1.75, confirming a slightly male predominance. MEMs are composed of both neuroectodermal and one or more mesenchymal neoplastic elements (1). Combining data from the Freitas et al. and the Nael et al. studies (14, 15), and our own observations, the most common mesenchymal element is ERMS, and less often other variants. The neuroectodermal elements may cover the entire spectrum of neuroblastic phenotypes, but they were predominantly GN and GC. Only sporadic cases have been reported with malignant peripheral nerve sheath tumour (MPNST) (7, 28).

In most cases, the malignant component consisted of the mesenchymal elements, as in our patient.

MEMs should be distinguished from another biphenotypic neuromuscular tumour, known as “benign triton tumour” or “neuromuscular choristomas”, which also contains neural tissue and skeletal muscle at varying levels of differentiation. The main difference would consist in the absence of malignant degeneration in benign triton tumours. However, VandenHeuvel reported the case of a 35-month-old girl affected by MEM, arising in association with benign triton tumour in the tongue. This finding can suggest that also triton tumours may have malignant potential and may have a possible relationship with MEMs (16).

Previous molecular studies performed on MEMs have revealed chromosomal changes and gene rearrangements. Though quite limited, the data reported in literature demonstrate remarkable overlapping characteristics between MEMs and RMS, including demographic features (male predominance, young age < 2 years), anatomic distribution and immunohistochemistry. In particular the retained H3K27me3 expression, reported in all the 7 cases described by Huang (23) suggest a closer relationship to RMS than MPNST, in which H3K27me3 expression is lost. Unfortunately, the major limitation of this study is the impossibility to perform H3K27me3 expression in our patient.

In the recent years, also cytogenetic abnormalities suggest that MEM might potentially constitute a variant of RMS (3, 6, 7, 29). For example, the *DICER1* mutation reported by Davidson and previously not described in MEM, has been described in ERMS as well as in RMS and in anaplastic sarcoma, consolidating the link between MEM and ERMS evidenced in the Huang report (23).

The latest edition of World Health Organization classification of soft tissue and bone tumours categorised MEM under “Skeletal Muscle Tumor” (30), while in the 2013 edition, they were classified as nerve sheath tumours. All these data suggest a stronger link to RMS than MPNST, as previously suggested by Kleinschimidt-DeMasters et al, who found that intracranial MEMs showed gene expression pattern similar to MPNST (28).

Nevertheless, due to the singularity of these tumours, the diagnostic criteria of MEM are not well defined and may be ambiguous; it is also challenging to determine prognostic factors and outcomes. Some MEMs have initially been diagnosed as pure RMS because the biphasic histological pattern characteristic of the MEM can be hardly detected in small biopsies (31).

Moreover, most cases showed a wide range of growth patterns, including myxoid-cellular areas, fascicular spindle cell and compact round cells, and immunomarkers of multiple lineages of differentiation, which complicate the diagnosis. Also in our case, the first diagnosis was ERMS, and the patient was treated according to the RMS protocols. However, after examining a larger amount of tissue obtained from the surgery, our final diagnosis was MEM.

This, along with other cases of MEMs, has taught us to be prudent in the histological diagnosis of MEM because biphasic, heterologous tumours may not be correctly identified when only small specimens are examined on routine hematoxylin and eosin-stained sections; ancillary studies may be required, such as immunohistochemistry, electron microscopy, or molecular analysis. It is supposed that the more undifferentiated the tumour is, the higher the probability of expressing markers of various differentiation lineages. Molecular data can even provide diagnostic accuracy in cases that are tricky to interpret.

It is well-known that, regarding soft tissue pathologies, molecular characterisation can contribute untangle the equivocal morphologic overlap between subtypes of sarcomas, especially when they arise in non-canonical anatomical sites (32).

Nevertheless, the results of molecular studies must always be contextualised in the context of an accurate histopathologic evaluation. In fact, a detected genetic mutation is not in itself a diagnosis, but merely a partial indicator.

Therefore, we think that the future diagnostic approach should routinely include molecular analysis and the use of bioinformatic mutation detection tools, in order to find peculiar gene rearrangements and mutations of MEMs. This will be integrative and supportive for the pathologist and fundamental in providing a better understanding of the disease aetiology of MEMs and in the search for new therapeutic targets and biomarkers.

General consensus for the management and therapeutic regimen for MEM seems to be a multimodal treatment strategy, including a combination of surgery and chemotherapy (7, 20). When the tumour is unresectable or the disease has metastasised in other sites, the prognosis is worse (15, 17). Considering the 5 patients lost at follow-up, our review revealed MEMs to have the same prognosis as other paediatric chemotherapy sensitive soft tissue sarcomas, with 82% (14/17) of children affected by MEM surviving with no evidence of disease (NED) following multimodality treatment approach.

## 6 Conclusions

MEMs have different and peculiar characteristics that distinguish them from other soft tissue sarcomas and an increased risk of delay in diagnosis. Despite the paucity of reported data, we emphasise the importance of an integrative morpho-molecular approach to support the diagnosis and understanding of the biology and behaviour of this rare and insidious tumour.

## Author contributions

FP: Conceptualization, Data curation, Formal analysis, Investigation, Methodology, Resources, Supervision, Validation, Visualization, Writing – original draft, Writing – review & editing. ET: Conceptualization, Data curation, Investigation, Methodology, Resources, Supervision, Validation, Writing – review & editing. FD: Data curation, Investigation, Methodology, Supervision, Validation, Writing – review & editing. AC: Data curation, Investigation, Methodology, Software, Supervision, Validation, Writing – review & editing. CR: Data curation, Investigation, Validation, Writing – review & editing. EA: Data curation, Investigation, Validation, Writing – review & editing. AL: Formal analysis, Investigation, Methodology, Supervision, Writing – review & editing. SA: Conceptualization, Data curation, Formal analysis, Investigation, Methodology, Project administration, Supervision, Validation, Visualization, Writing – review & editing. FF: Conceptualization, Data curation, Formal analysis, Investigation, Methodology, Project administration, Supervision, Validation, Visualization, Writing – review & editing.
